# Longitudinal evaluation of workflow optimization in radiotherapy: A 4‐year retrospective study

**DOI:** 10.1002/acm2.70252

**Published:** 2025-08-31

**Authors:** Chenlei Guo, Peng Huang, Bining Yang, Hao Jing, Wenlong Xia, Zhuanbo Yang, Kuo Men, Jianrong Dai

**Affiliations:** ^1^ Department of Radiation Oncology, National Cancer Center/National Clinical Research Center for Cancer/Cancer Hospital Chinese Academy of Medical Sciences and Peking Union Medical College Beijing China

**Keywords:** artificial intelligence, quality assurance, radiotherapy, treatment efficiency, workflow optimization

## Abstract

**Background:**

Efficient workflows are essential for timely, high‐quality radiotherapy. In 2020, an internal audit identified key workflow bottlenecks, including long patient wait times, suboptimal treatment planning, and inadequate quality control. Accordingly, since early 2021, we implemented targeted workflow improvements to address these deficiencies.

**Purpose:**

This retrospective study evaluated the impact of workflow optimization from 2021 to 2024, focusing on operational and clinical metrics.

**Methods:**

The radiotherapy workflow includes patient admission, imaging (CT/MR simulation), treatment planning, multi‐level quality assurance, plan transfer, and first radiation delivery. Optimization measures included AI‐assisted tumor contouring, standardized planning protocols, dynamic scheduling, automated quality control, and predictive maintenance using machine learning. Data from 25 838 patients were analyzed, examining wait times, planning durations, and quality control efficiency.

**Results:**

The total admission‐to‐treatment duration decreased by 30%, from 12.83 days (interquartile range [IQR] = 9.48–16.98) pre‐2020 to 8.78 days (IQR = 3.72–10.48) in 2021, stabilizing at 9.34 days (IQR = 4.27–12.01) by 2024. CT/MR simulation preparation time increased from 2.24 days (IQR = 1.24–2.17) to 3.82 days (IQR = 1.47–4.65). Treatment planning preparation time decreased significantly from 5.75 days (IQR = 3.85–7.02) to 2.57 days (IQR = 1.48–3.42). Treatment planning duration also decreased from 2.68 days (IQR = 2.07–2.87) to 1.33 days (IQR = 0.91–1.84). A new physics review stage, introduced in 2021, required 0.04 days (IQR = 0.01–0.04) in 2024. Clinical review time decreased from 0.78 days (IQR = 0.38–0.98) pre‐2020 to 0.46 days (IQR = 0.06–0.80) in 2024. Plan transfer time reduced from 0.42 days (IQR = 0.11–0.66) to 0.24 days (IQR = 0.05–0.28). The preparation time for the first radiation delivery decreased from 2.48 days (IQR = 1.84–3.27) to 0.81 days (IQR = 0.22–1.06).

**Conclusion:**

Workflow optimization yielded sustained improvements in radiotherapy efficiency, safety, and patient satisfaction, highlighting the impact of AI‐driven tools, standardized workflows, and data‐driven management in advancing global radiotherapy practices.

## 1 INTRODUCTION

Radiotherapy is a cornerstone of modern cancer treatment, with approximately 60% of patients with cancer requiring it at some stage during their care.^1^, ^2^ However, its effectiveness inherently depends on workflow efficiency, involving processes from imaging acquisition to final treatment delivery.^3^ Previous studies, including that by Guo et al.,^4^ have identified workflow inefficiencies—particularly delays in module transitions, such as the waiting period between computed tomography (CT) simulation and treatment planning—as critical bottlenecks. These delays compromise treatment timeliness, heighten patient anxiety, and increase the risk of tumor progression.^4^ Key challenges, such as prolonged waiting times, suboptimal treatment planning efficiency, and insufficient quality assurance (QA) protocols, continue to hinder the timely and effective delivery of care.^5^ These inefficiencies are particularly concerning, given the increasing global burden of cancer, which is expected to drive a significant increase in the demand for radiotherapy in the future.^6^, ^7^, ^8^

In 2020, our department analyzed the real‐time clinical workflows of 17 620 patients (an initial audit cohort) with cancer and identified several critical bottlenecks.^4^ Our internal analysis identified major delays in tumor contouring and resource allocation, particularly in head and neck tumor treatment, which we attributed to the complexity of anatomical structures and uneven workload distribution. These findings are consistent with those of previous studies, which highlighted similar workflow inefficiencies in radiotherapy departments.^9^, ^10^

Furthermore, previous research^11^ has indicated that equipment downtime further exacerbates these delays, reinforcing the necessity of implementing proactive maintenance strategies. Collectively, these inefficiencies underscore the imperative for workflow optimization to improve treatment efficiency and patient outcomes.

Notably, emerging technologies, particularly artificial intelligence (AI), have demonstrated considerable potential in addressing these challenges.^12^ AI‐assisted contouring tools have significantly reduced the time required and inter‐observer variability associated with manual tumor delineation.^13^, ^14^, ^15^ Recent evaluations of AI‐based auto‐contouring solutions have demonstrated high accuracy across multiple anatomical regions, further supporting their clinical integration.^16^ Additionally, integrating AI‐driven solutions can streamline workflows, reduce bottlenecks in critical care processes, and enhance the efficiency of treatment planning and resource allocation.^17^ These advancements hold promise for improving radiotherapy workflows and optimizing treatment delivery.

In addition to contouring, adaptive radiotherapy workflows, which incorporate imaging‐driven real‐time adaptation, have been increasingly implemented to enhance precision and workflow efficiency.^18^ Recent work on machine‑learning scheduling has further boosted planning throughput and resource utilization.^19^, ^20^ Department‑wide workflow platforms, such as PlanQ, apply these algorithms in real time, streamlining task coordination and cutting planning‑coordination workload by about 70%, thereby improving the overall efficiency and scalability.^21^ Similarly, innovative risk assessment tools can be used to reduce clinic overspill appointments and streamline scheduling, providing insights into the broader application of such technologies in optimizing healthcare workflows.^22^

Moreover, discrete‐event simulation‐based scheduling studies have shown that a hybrid pull–push scheduling strategy can effectively reduce average waiting times and improve treatment timeliness.^23^ Similarly, predictive maintenance strategies using long short‐term memory (LSTM) networks have proven effective in reducing equipment downtime and improving system reliability.^24^, ^25^ Despite these advancements, empirical evidence regarding their long‐term impact on clinical workflows remains limited, underscoring the need for additional longitudinal studies.

Therefore, our department initiated a series of workflow optimizations in 2021 to address these challenges. These included AI‐assisted tumor and OAR contouring to streamline segmentation and enhance consistency, standardized planning protocols to reduce inter‐plan variability, automated quality control systems to enable real‐time verification of treatment plans and records, predictive maintenance algorithms based on LSTM models to reduce equipment downtime, and dynamic resource scheduling strategies to improve patient throughput and reduce bottlenecks. A detailed description of the timing and implementation of these interventions is provided in the following section.

Specifically, this study aimed to assess the long‐term impact of these interventions over 4 years (2021–2024) by analyzing operational and clinical metrics from a cohort of 25 838 patients. By integrating technological advancements and process optimizations, this investigation contributes to the growing body of evidence supporting the use of AI‐driven solutions in radiotherapy workflows.^15^, ^26^ Furthermore, in this study, we discuss how these interventions can be adapted for resource‑constrained centers, acknowledging that their success depends on both technological tools and sufficient trained personnel, which is crucial given the increasing global burden of cancer.^7^, ^18^, ^27^

## 2 METHODS

### 2.1 Study design

This retrospective observational study quantified the time required for patients to progress through the radiotherapy workflow, from admission to treatment initiation, over 4 years (January 2021 to December 2024). Since the treatment duration varies widely among patients, data from the post‐initiation treatment phase were excluded. All contemporary radiotherapy techniques were represented in the patient cohort; treatments ranged from 3D conformal radiotherapy (3DCRT) to intensity‐modulated radiotherapy (IMRT), and volumetric‐modulated arc therapy (VMAT), mirroring routine practice over the 4‐year period. Patients were not excluded on the basis of plan modality or complexity. Urgent or emergency courses were likewise included, as our objective was to characterize real‐world workflow performance across the full spectrum of case types. Ultimately, our 2021–2024 study included 25 838 first‐time radiotherapy patients with head and neck, thoracic, and abdominal tumors.

### 2.2 Workflow definition and time points

**FIGURE 1:**
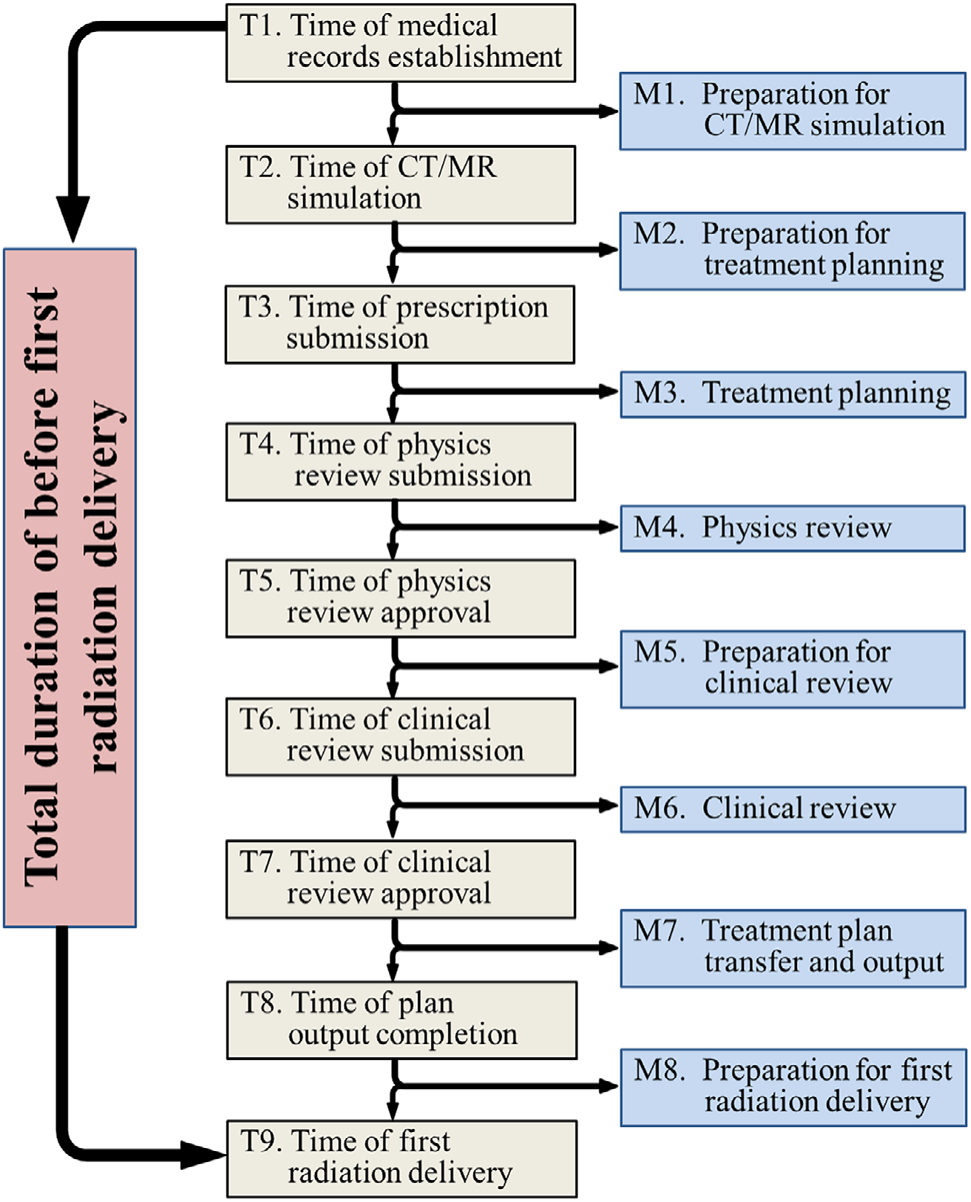
Workflow stages in the radiotherapy process (T1–T9). This figure illustrates the sequential workflow modules (M1–M8) in radiotherapy, from admission (T1) to first radiation delivery (T9). Key stages include preparation for imaging, treatment planning, multi‐level quality assurance, and final treatment setup. Each module is defined by critical milestones recorded in the MOSAIQ radiotherapy information system. Arrows indicate transitions between modules, highlighting areas optimized through automation and standardization. (CT, computed tomography; MR, magnetic resonance).

Nine critical time points (T1–T9) were defined to evaluate the radiotherapy workflow systematically, each corresponding to one of eight sequential workflow modules (M1–M8). Figure 1 illustrates the sequence of these modules and their interconnections. The time points were defined as follows: T1: time of medical records establishment; T2: time of CT/magnetic resonance (MR) simulation; T3: time of prescription submission; T4: time of physics review submission; T5: time of physics review approval; T6: time of clinical review submission; T7: time of clinical review approval; T8: time of plan output completion; and T9: time of first radiation delivery.

The workflow modules were categorized as follows: M1: preparation for CT/MR simulation; M2: preparation for treatment planning; M3: treatment planning; M4: physics review; M5: preparation for clinical review; M6: clinical review; M7: treatment plan transfer and output; and M8: preparation for first radiation delivery.

At the time of CT/MR simulation (M1), only a preliminary treatment intent was provided by the attending physician. A formal treatment prescription was generated during M2, after the acquisition of simulation imaging, based on delineated targets and organs‐at‐risk (OARs), and was finalized during multidisciplinary ward rounds. Multidisciplinary ward rounds were conducted weekly to confirm treatment intent and target delineation during M2 and perform clinical review and plan approval during M6. Additional small‐scale sessions may be convened for urgent cases to ensure timely workflow progression. Following plan finalization at the end of M7, a portion of the M8 stage includes physics‐led patient‐specific quality assurance (QA). According to departmental policy, all hypo‐fractionated treatments (dose per fraction >4 Gy) require QA completion prior to the first treatment, whereas conventional fractionation plans must undergo QA within the first three treatment fractions.

All time points were automatically logged in the MOSAIQ radiotherapy information management system (Elekta)^28^ via the following predefined workflow status tags to ensure real‐time tracking: Prepare,″ Schedule,″ and Complete.″ Workflow statuses were manually updated by responsible personnel, including physicians, physicists, and therapists (Figure 1).

### 2.3 Three‐tier review system

A three‐tier review system, comprising a physics review, clinical review, and ward rounds, was established to ensure the accuracy and safety of the radiation treatment plan. Compared with the conventional two‐tier review process described by Sack et al.,^29^ which primarily emphasizes technical verification and clinical compliance, our three‐tier review system integrates an additional multidisciplinary review during ward rounds. This additional layer was introduced to allow a more thorough evaluation of clinical and technical parameters, with the expectation of reducing errors and enhancing interprofessional collaboration.

During the physics review, the assigned physicist designed and finalized the initial treatment plan.^30^ A senior physicist subsequently reviewed the plan to verify technical parameters, dose distributions, and overall design accuracy. Upon passing the physics review, the clinical supervising physician assessed the plan's compliance with the prescribed clinical objectives and dose constraints under clinical review. Following both physics and clinical reviews, the final plan was approved during ward rounds, ensuring interdisciplinary validation. Specifically, this process involved the entire clinical team to ensure that the plan met all clinical and technical standards before proceeding to patient treatment. This three‐level review system provided a layered QA mechanism to minimize errors and ensure safe and effective treatment.

### 2.4 Stage‐wise workflow improvements (2021–2024)

Following the publication of the initial workflow analysis,^4^ our department prioritized comprehensive process oversight with a strong emphasis on reducing patient wait times. We standardized timestamp recording for all key events using the MOSAIQ system (Elekta); introduced strict turnaround benchmarks for physicians, physicists, and technologists; and fostered a department‐wide culture that emphasized minimizing the interval from patient admission to first irradiation, to reduce treatment delay and patient anxiety, without compromising plan quality.

The concrete improvements implemented in each workflow module are summarized below:

#### 2.4.1 M1 (Preparation for CT/MR simulation)

This is the interval from patient admission (T1) to completion of simulation imaging (T2). To shorten this interval, two major measures were introduced at the end of 2020. First, the MOSAIQ system was configured to generate automatic reminders for each newly admitted patient, and weekly compliance audits were established to ensure timely appointment scheduling. Second, the simulation technologist team was expanded from 12 to 18 members, and staffing schedules were adjusted to better accommodate peak‐hour demand, with the aim of improving access to simulation appointments.

#### 2.4.2 M2 (Preparation for treatment planning)

This stage spans contouring, image registration, and prescription finalization after simulation (T2 → T3). Beginning in early 2021, we integrated AI‐driven auto‐segmentation tools,^31^, ^32^ initially for delineating OARs across multiple tumor sites. Between 2022 and 2023, these tools were further enhanced to support automatic image registration^33^ and AI‐based target volume (GTV and CTV) delineation^34^ for a broader range of anatomical regions. These advances were intended to shorten contouring times, improve inter‐observer consistency, and reduce variability in pre‐planning workflows, thereby facilitating the transition to treatment planning.

#### 2.4.3 M3 (Treatment planning)

This is the process of generating and optimizing the treatment plan within the TPS (T3 → T4). In parallel with M2, we fully implemented the standardized planning protocols in early 2021, including unified dose–volume constraints, optimization objectives, and beam arrangement templates.^35^ A semi‐automated dose calculation workflow^36^ was also established, with the aim of reducing planning time and improving overall planning efficiency.

#### 2.4.4 M4 (Physics review)

This intermediate technical QA stage occurs between plan creation and clinical approval (T4 → T5). Consistent with the institutional emphasis on timeliness described in M1, a departmental shift in both culture and policy was adopted starting in 2021 to improve the review efficiency across all stages. A dedicated physics review process (M4) was introduced to verify technical parameters and dose distributions prior to clinical approval.^30^ To further streamline this step, we developed the treatment plan review system (TPRS, “AutoReview”^37^), which automates key checklist items for both physics (M4) and clinical (M6) reviews; the system was designed to markedly increase review throughput and enhance anomaly detection, as shown in prior internal validation.

#### 2.4.5 M5 (Preparation for clinical review)

This brief queue period bridges physics approval and clinical sign‑off (T5 → T6). Automated notification workflows and a dashboard of pending items were implemented to expedite communication among clinicians, physicists, and technologists, thereby reducing delays in clinical‑review preparation.

#### 2.4.6 M6 (Clinical review)

This multidisciplinary review finalizes plan approval before export (T6 → T7). Standing (virtual) ward rounds were scheduled, and concise AutoReview summary reports were provided to supervising physicians to support rapid verification of clinical objectives and timely final approval.

#### 2.4.7 M7 (Treatment‑plan transfer and output)

This stage covers plan export to the record‑and‑verify system and preparation of patient‑specific QA documentation (T7 → T8). Consistent with our emphasis on efficiency, we scripted a batch‑export utility, increased medical‑physicist staffing from 42 to 58, and upgraded network bandwidth to shorten file‑transfer times. Automated notification systems further facilitated seamless hand‑offs among physicians, physicists, and technologists.

#### 2.4.8 M8 (Preparation for first radiation delivery)

This final step spans patient‑specific QA, machine scheduling, and initiation of the first treatment fraction (T8 → T9). Interventions included commissioning two additional linear accelerators (expanding capacity from seven to nine units), adopting a dynamic scheduling algorithm with predictive‑maintenance inputs to mitigate machine‑time bottlenecks, and integrating ArcherQA,^38^ a GPU‑accelerated Monte‑Carlo prescreening tool introduced in 2022 to flag dose discrepancies before physical QA. These resource expansions and system‑level optimizations were intended to enhance operational throughput and improve timely patient access to radiotherapy.

### 2.5 Data collection

**FIGURE 2:**
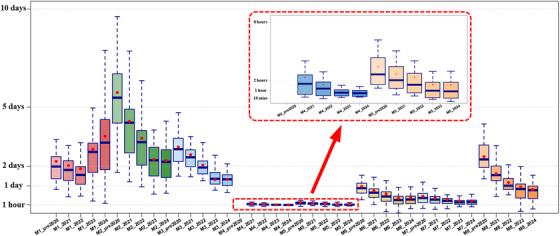
Annual trends in radiotherapy workflow durations (pre‐2020 to 2024). This figure illustrates the progression of workflow durations across different modules (M1–M8) from pre‐2020 to 2024, highlighting the effects of workflow optimizations. The box plots represent the distribution of workflow durations, where boxes, horizontal lines, and red markers denote interquartile range (IQR: 25%–75%), median, and mean values, respectively. Whiskers extend to capture variability beyond the IQR, depending on the data distribution. The inset plot highlights M4 (Physics review) and M5 (Preparation for clinical review), which were introduced into the workflow in 2021, resulting in notable efficiency improvements. M4 was not part of pre‐2020 workflows, explaining its exclusion from earlier data.

Workflow time data were automatically logged and extracted from the MOSAIQ system. For each workflow stage we measured the full elapsed time from the completion of one module to the start of the next. These stage‑to‑stage intervals are the values plotted in Figure 2, covering both active work and any waiting. The overall admission‑to‑treatment duration is the sum of all such intervals from the first event (T1) to the first treatment fraction (T9).

In parallel, patient experience was assessed with an anonymous five‑item Likert survey completed at treatment completion and again at the first routine follow‑up; responses were entered into REDCap and summarized annually. Plan‑revision frequency was captured automatically by the treatment‑planning system, which logs every modification request and approval; audit files were exported quarterly for quality review.

### 2.6 Statistical analysis

Descriptive statistics, including the mean, median, minimum, maximum, and interquartile range (IQR), were computed for each time point and workflow module. Data normality was assessed using the Kolmogorov–Smirnov (K–S) test, as all analyzed sample sizes exceeded 50 (n ≥ 50). Since the normality tests indicated that most of the data did not follow a Gaussian distribution (*p* < 0.05), non‐parametric methods were used for statistical comparisons. Between‐group differences were evaluated using the Mann–Whitney U test for two‐group comparisons and the Kruskal–Wallis H test for multiple‐group comparisons. To account for multiple comparisons and reduce Type I error, Holm–Bonferroni correction was applied where necessary. All statistical analyses were performed using IBM SPSS Statistics for Windows, version 27.0 (IBM Corp.). The significance threshold was set at a *p*‐value of < 0.05 following correction for multiple comparisons.

### 2.7 Exclusion criteria and bias assessment

This study exclusively included patients undergoing first‐time radiotherapy and excluded those undergoing multi‐course treatments. The rationale for exclusion was two‐fold: (1) multi‐course patients may bypass or omit some workflow steps, leading to incomplete data for process evaluation, and (2) first‐time patients provide a clearer representation of workflow efficiency improvements as they undergo the entire radiotherapy process. Additionally, post‐treatment workflow data were excluded owing to substantial variability in treatment regimens among patients. Potential selection bias was assessed by comparing demographic characteristics (including age, sex, and tumor type) between the excluded and included patients using Chi‐square and Mann–Whitney U tests (*p* < 0.05). Resampling techniques confirmed that the exclusion criteria did not significantly affect key operational metrics, including workflow durations. As the excluded patients comprised <5% of the cohort and exhibited demographic characteristics comparable to those of the included patients, the study findings remain robust and generalizable.

## 3 RESULTS (WITH PRELIMINARY DISCUSSION)

### 3.1 Statistical overview

Table 1 presents the summary statistics for each workflow module, and Figure 2 shows inter‐year variability and overall workflow trends. The durations for all workflow modules (M1–M8) were measured in days with two‐decimal precision and are reported as mean, median, and IQR (25%–75%). All calculations excluded weekends and public holidays to ensure data consistency. Within each workday, time was tracked continuously on a 24‐h basis, with no end‐of‐day cutoff applied. A recorded duration of 0.00 indicates that the process was completed in less than 10 min.

**TABLE 1:** Summary of workflow durations across workflow modules (between pre‐2020 and 2021–2024).

Module	Pre‐2020	2021	2022	2023	2024	Mean time diff. (pre‐2020→2024)	Key interventions
**M1**	2.24/1.72 (1.24–2.57)	2.03/1.59 (1.17–2.36)	1.86/1.25 (1.02–2.01)	2.86/2.58 (1.39–3.27)	3.52/2.88 (1.47–4.65)	+1.28	CT reminder system and staffing and schedule Adjustments
**M2**	5.75/5.22 (3.85–7.02)	4.28/4.15 (2.18–5.10)	3.43/3.02 (1.73–3.95)	2.33/2.28 (1.24–3.17)	2.29/2.15 (1.18–3.02)	−3.46	AI‐assisted tumor/OAR contouring
**M3**	2.98/2.75 (2.07–3.57)	2.59/2.31 (1.87–2.92)	2.05/1.81 (1.54–2.34)	1.31/1.40 (1.02–1.77)	1.33/1.34 (0.91–1.64)	−1.65	Standardized protocols and semi‐auto calculation workflow
**M4**	Null	0.10/0.04 (0.02–0.11)	0.07/0.03 (0.02–0.08)	0.04/0.03 (0.02–0.05)	0.04/0.02 (0.01–0.04)	N/A	TPRS AutoReview system
**M5**	0.14/0.08 (0.06–0.20)	0.11/0.06 (0.05–0.16)	0.10/0.03 (0.03–0.12)	0.07/0.01 (0.01–0.08)	0.07/0.01 (0.01–0.08)	−0.07	Standardized protocols and TPRS AutoReview system
**M6**	0.98/0.78 (0.58–1.18)	0.75/0.55 (0.36–0.88)	0.59/0.32 (0.18–0.73)	0.37/0.19 (0.05–0.51)	0.40/0.20 (0.05–0.50)	−0.58	TPRS AutoReview system
**M7**	0.47/0.30 (0.11–0.66)	0.38/0.21 (0.08–0.49)	0.30/0.17 (0.07–0.37)	0.23/0.10 (0.04–0.27)	0.24/0.11 (0.05–0.28)	−0.23	Staffing and linac expansion
**M8**	2.48/2.21 (1.84–3.27)	1.62/1.47 (1.20–2.14)	1.17/0.80 (0.84–1.45)	0.95/0.83 (0.27–1.15)	0.81/0.76 (0.22–1.06)	−1.67	Staffing and linac expansion and ArcherQA tool

*Note*: Time durations for all workflow modules (M1–M8) were measured in days with two‐decimal precision and reported as mean/median and interquartile range (IQR: 25%–75%). This table compares the descriptive statistics of workflow durations for modules M1–M8 across five time periods: pre‐2020 (baseline), 2021, 2022, 2023, and 2024 — together with the mean time difference between baseline and 2024; positive values indicate longer processing time and negative values indicate a reduction. Data are presented as mean, median, and IQR (25%–75%). Durations are measured in days, excluding weekends and public holidays. Pre‐2020 data are excluded for M4 (physics review) owing to its absence before 2021. Key interventions implemented for each module are summarized in the right‑hand column.

Significant reductions in workflow durations were observed from 2021 onward compared to pre‐2020 workflows (Table 1 and Figure 2). Note: In the pre‐2021 workflow (as described in Guo et al.^4^), M3 included treatment planning as well as verification steps that are now represented separately as M3, M5, M6, and M7. A dedicated physics review module (M4) did not exist and is therefore absent from pre‐2020 data. M4 was formally introduced in 2021 as part of a standardized three‐tier QA system to enable targeted technical checks before clinical review, without increasing the overall workflow duration.

### 3.2 Overall workflow duration

Total time from patient admission to first radiation delivery decreased substantially following the implementation of workflow optimizations beginning in 2021 (Table 1, Figure 2). The median total workflow duration dropped by approximately 20% in the first year, from ∼13.1 days pre‐2020 to ∼10.4 days in 2021. A further reduction was observed in 2022 (7.4 days), after which the duration stabilized at around 7.3–7.5 days in 2023–2024 (overall *p* < 0.001). This pattern suggests that the majority of efficiency gains were realized within the first 2 years of intervention, with improvements sustained thereafter. Notably, even in 2024, the total duration remained nearly 5 days shorter than the baseline, highlighting the lasting impact of the optimization efforts.

In addition to median reductions, the upper bound of the workflow distribution also shifted significantly: the time required for 95% of patients to complete the workflow (M1–M8) decreased from 17 days pre‐2020 to approximately 11 days between 2021 and 2024. This sustained improvement underscores the long‐term impact of system‐wide enhancements, including AI‐assisted planning, automated QA, and structured scheduling protocols.

### 3.3 Module‐specific improvements

#### 3.3.1 M1 (Preparation for CT/MR simulation)

The duration of M1 initially declined following the 2021 optimization, with median values dropping from 1.59 days in 2021 to 1.25 days in 2022 (Table 1, Figure 2). However, this improvement was not sustained. In 2023 and 2024, M1 duration increased markedly, reaching 2.58 and 2.88 days, respectively, exceedingly even pre‐2020 levels. These changes were statistically significant across all years (overall *p* < 0.001). This trend suggests that early gains from workflow enhancements were eroded over time, and new operational bottlenecks have since emerged in the simulation stage.

#### 3.3.2 M2 (Preparation for treatment planning)

The duration of M2 showed a progressive and statistically significant reduction from 2021 to 2023, following the introduction of AI‐assisted contouring and workflow standardization (Table 1, Figure 2). Median M2 duration decreased from 5.22 days pre‐2020 to 4.15 days in 2021, then to 3.02 days in 2022, and further to 2.28 days in 2023 (overall *p* < 0.001). In 2024, the duration remained stable at 2.45 days (*p* = 0.412 compared to 2023), indicating that recent improvements were maintained. These reductions highlight the impact of automation and process refinement on treatment planning efficiency, especially in a previously time‐intensive workflow module.

#### 3.3.3 M3 (Treatment planning)

Treatment planning time (M3) demonstrated a steady and significant decline over the study period (Table 1, Figure 2). The median duration decreased from 2.75 days pre‐2020 to 2.31 days in 2021, further dropping to 1.81 days in 2022 and stabilizing around 1.34 days in 2023–2024 (overall *p* < 0.001). These findings reflect the impact of standardized planning templates and semi‐automated dose calculation workflows, which contributed to consistent gains in planning efficiency.

#### 3.3.4 M4 (Physics review)

The physics review stage (M4) was formally introduced in 2021 as part of a three‐tier QA system and did not exist prior to that. From its introduction, the median time required for physics review decreased from 0.04 days in 2021 to 0.02 days in 2024 (*p* = 0.481). Although the absolute time remained low throughout, the consistent reduction and stable integration of M4 demonstrate its efficiency and minimal impact on total workflow time while improving quality assurance (Table 1, Figure 2).

#### 3.3.5 M5–M6 (Clinical review preparation and clinical review)

Both preparation for clinical review (M5) and the review itself (M6) showed progressive and significant reductions in duration from pre‐2020 through 2023, with stabilization in 2024 (overall *p* < 0.001 for both modules). Median durations dropped from 0.78 days to 0.20 days, indicating a ∼70% improvement. These improvements can be attributed to the implementation of automatic notification systems and the AutoReview platform, which streamlined communication and reduced delays in interdisciplinary plan evaluation (Table 1, Figure 2). AutoReview accelerates the process so that roughly sixty plans can be checked in the time a single manual review once required, while also boosting anomaly detection by 19.2%.

#### 3.3.6 M7 (Treatment plan transfer and output)

M7 duration also significantly declined over the study period (Table 1, Figure 2), from a median of 0.30 days pre‐2020 to 0.11 days in 2024 (*p* < 0.001). The most substantial reductions occurred between 2021 and 2023, after which the duration plateaued. These gains reflect reduced physicist workload and improved handover efficiency following infrastructure expansion and clearer role delineation in plan finalization.

#### 3.3.7 M8 (Preparation for first radiation delivery)

M8 was a critical bottleneck identified in the 2020 institutional workflow analysis,^4^ and targeted interventions since then have yielded substantial improvements (Table 1, Figure 2). The median duration decreased from 2.21 days pre‐2020 to 1.47 days in 2021, then to 0.80 days in 2022, and further to 0.76 days in 2024 (overall *p* < 0.001). These reductions reflect the impact of streamlined scheduling, automated coordination tools, and interdepartmental workflow alignment. By 2024, over 90% of patients were prepared for first radiation within 1.5 days, compared to 3–4 days before 2020. This sustained gain underscores the clinical value of operational efficiency in treatment initiation.

### 3.4 Tumor‐specific analysis of M2 and M3 duration

**FIGURE 3:**
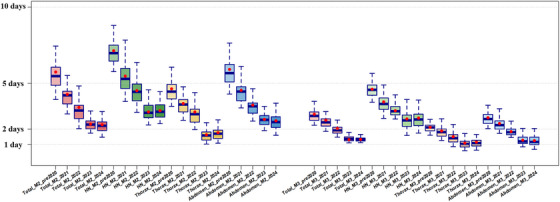
Annual trends in M2 and M3 workflow durations across tumor sites (pre‐2020 to 2024). This figure illustrates the progressive reductions in M2 (Preparation for treatment planning) and M3 (Treatment planning) durations across different tumor sites from pre‐2020 to 2024. HN” represents the head‑and‑neck groups. The box plots represent the distribution of workflow durations, where boxes, horizontal lines, and red markers indicate interquartile range (IQR: 25%–75%), median, and mean values, respectively. Whiskers extend to capture variability beyond the IQR, depending on the data distribution.

To address the workflow inefficiencies and site‐specific delays highlighted by Guo et al.^4^ and investigate the impact of workflow optimizations across different anatomical regions, we analyzed M2 and M3 durations separately for the “head and neck” (including NKT, NPC, CNS, and brain metastases), “thorax” (including lung, esophagus, breast, and lymph node), and “abdomen” (including rectum, liver, prostate, gynecologic, and extremities) groups (Table 2, Figure 3). All three groups demonstrated consistent and statistically significant reductions over time (Kruskal–Wallis test, *p* < 0.001), although baseline durations and absolute improvements varied across sites.

**TABLE 2:** Time durations of modules M2 and M3 and tumor sites.

	Total		H and N		Thorax		Abdomen	
	M2	M3	M2	M3	M2	M3	M2	M3
**Pre‐2020**	**5.75/5.22 (3.85–7.02)**	**2.98/2.75 (2.07–3.57)**	**7.15/6.82 (5.40–8.61)**	**4.62/4.54 (3.28–5.72)**	**4.65/4.27 (3.14–5.81)**	**2.12/2.10 (1.26–2.67)**	**5.92/5.43 (4.10–7.22)**	**2.75/2.58 (1.78–3.61)**
**2021**	**4.28/4.15 (2.18–5.10)**	**2.59/2.31 (1.87–2.92)**	**5.48/5.12 (3.32–7.51)**	**3.79/3.51 (2.67–4.83)**	**3.68/3.55 (1.88–4.72)**	**1.88/1.73 (0.98–2.41)**	**4.55/4.38 (2.34–5.48)**	**2.37/2.15 (1.56–3.06)**
**2022**	**3.43/3.02 (1.73–3.95)**	**2.05/1.81 (1.54–2.34)**	**4.53/4.37 (2.33–6.26)**	**3.21/3.14 (2.34–4.31)**	**3.13/2.84 (1.33–3.85)**	**1.51/1.30 (0.74‐2.03)**	**3.58/3.42 (1.95–4.13)**	**1.84/1.76 (1.28–2.48)**
**2023**	**2.33/2.28 (1.24–3.17)**	**1.31/1.40 (1.02–1.77)**	**3.13/3.02 (1.94–4.88)**	**2.67/2.48 (1.28–3.68)**	**1.63/1.53 (0.54–2.37)**	**1.08/0.98 (0.38–1.57)**	**2.64/2.58 (1.48–3.62)**	**1.28/1.11 (0.91–2.06)**
**2024**	**2.29/2.15 (1.18–3.02)**	**1.33/1.34 (0.91–1.64)**	**3.22/3.05 (2.01–4.73)**	**2.73/2.64 (1.31–3.75)**	**1.77/1.63 (0.65–2.43)**	**1.15/1.01 (0.55–1.64)**	**2.55/2.38 (1.32–3.58)**	**1.25/1.06 (0.79–2.15)**

*Note*: All data are calculated in days with two decimal places remaining and displayed in the form of mean/median (25%, 75%). This table compares the descriptive statistics of workflow durations for M2 (Preparation for treatment planning) and M3 (Treatment planning) across different tumor sites. Data are presented as mean, median, and interquartile range (25%–75%). Durations are measured in days, excluding weekends and public holidays. Pre‐2020 data are excluded for M4 (physics review) owing to its absence before 2021.

In the head and neck (H&N) group, M2 duration decreased from a median of 6.82 days pre‐2020 to 3.15 days in 2024, while M3 declined from 4.54 to 2.64 days. In the thoracic group, M2 duration dropped from 4.27 to 1.73 days, and M3 from 2.10 to 1.01 days. In the abdominal group, M2 was reduced from 5.43 to 2.38 days, and M3 from 2.58 to 1.06 days.

These reductions reflect the broad effectiveness of workflow optimization strategies across anatomical regions. In H&N tumors, despite their anatomical complexity, substantial gains were achieved through AI‐assisted contouring and workload redistribution. For thoracic and abdominal cases, improvements were largely driven by standardized planning protocols and automated multi‐organ segmentation, which reduced manual editing time and improved delineation consistency.

## 4 DISCUSSION

This 4‑year retrospective evaluation shows that integrating AI‑assisted contouring, standardized workflows, and predictive analytics markedly improved radiotherapy efficiency, shortened treatment‑planning times, and increased patient satisfaction and safety compared with the pre‑2020 baseline. The streamlined process also supports stronger quality assurance and operational reliability.

### 4.1 System‐wide gains in workflow efficiency

Internal audit records show that the mean time from admission to first radiation fell by 31.6%, dropping from 12.83 days before 2020 to 8.78 days in 2021. This shorter interval remained largely stable through 2023 and rose only slightly to 9.34 days in 2024, a change that coincided with higher patient volume and new clinical services. The sustained improvement confirms the value of the 2021 module‑specific upgrades in M2, M3, and M8, together with the broader measures introduced in late 2020 such as unified time‑stamping, explicit turnaround targets, and a department‑wide focus on timeliness.

### 4.2 Addressing the challenges in CT/MR simulation (M1)

The rise in M1 time during 2023 and 2024 had two main causes. First, retirement of one CT simulator cut imaging capacity in half and lengthened queues. Second, wider use of deep inspiration breath hold (DIBH) for thoracic and left‑sided breast cases added coaching and set‑up time. A replacement simulator is scheduled for installation and is expected to shorten M1 by about 20% to 30%. Further reductions should come from better DIBH scheduling and guided respiratory‑training software. Together these steps are intended to return M1 to its earlier level and preserve long‑term efficiency.

### 4.3 AI‐driven improvements in M2 across tumor types

AI‑assisted contouring and standardized protocols cut M2 times across every tumor group. In head‑and‑neck cases, which have the most complex anatomy, the median dropped from 7.15 days before 2020 to 3.22 days in 2024, a reduction of about 4 days that accounts for 46.7% of the total workflow improvement. Thoracic plans fell from 4.65 days to 1.77 days over the same interval, contributing 34.5% of the overall time saving. Abdominal cases improved from 5.92 days to 2.55 days, a 3.5‑day gain that represents 40.3% of the reduction. These figures underline how automated delineation and multi‑organ segmentation lessen manual workload, improve consistency, and speed planning, especially in anatomically intricate regions.

### 4.4 Eliminating bottlenecks in first radiation delivery preparation (M8)

Preparation for the first radiation fraction fell from 2.48 days in 2020 to 0.72 days in 2021 and has remained under one day through 2024, a 71% improvement that exceeds earlier reports such as Williams and Drinkwater.^5^ Key contributors were automated scheduling, real‑time resource allocation, and predictive maintenance for the linear accelerators, all of which cut downtime, balanced staffing, and reduced delays. These measures have kept start‑up times low even as patient numbers and advanced‑planning techniques have risen. (Any product names cited in the Methods are given solely for reproducibility and do not imply endorsement.)

### 4.5 Most impactful gains and interventions

Table 1 indicates that two stages delivered the bulk of our overall improvement. M2 (Preparation for treatment planning) fell by 3.46 days after the introduction of AI‑assisted contouring together with unified dose‑constraint templates. M8 (Preparation for first irradiation) shortened by 1.67 days once automatic scheduling, predictive linac maintenance, and two additional accelerators were in place. These two modules together account for roughly 70% of the total reduction in admission‑to‑treatment time, marking them as the highest‑yield targets for centers aiming to improve plan turnaround with limited resources.

### 4.6 Comparison with other studies

The 45% and 59% reductions we observed in M2 and M3 are larger than the 40%–45% improvements reported by Sibolt et al.^39^ and Jiang et al.,^40^ We attribute the added benefit to the combined use of AI contouring, predefined dose constraints, beam templates, semi‑automated optimization, and real‑time scheduling. In line with Meyer et al,^16^ the AI tool is also expected to improve inter‑observer consistency, but our data show that efficiency gains depend on the full, standardized workflow, not the contouring software alone.

Quality control and patient‑centered outcomes improved in parallel. A three‑tier review introduced in 2021 cut plan‑revision requests by 20%, echoing the advantages reported by Sack et al ^29^ and the dosimetric benefits noted by Yan et al.^30^ The physics review now takes about 0.04 days despite the extra checkpoint, helped by AutoReview, which enhances throughput sixty‑fold. Faster preparation correlated with higher patient satisfaction: 92% of respondents were positive in 2021–2024 versus 75% before 2020, consistent with the link between timeliness and trust described by Sibolt  et al.^39^ Shorter lead times are also likely to strengthen schedule adherence, an effect shown in studies of timely radiotherapy initiation.^40^

Maintaining these gains will be challenging as volume grows. The slight rise in processing time during 2024 mirrors the workload escalation identified by Tohyama et al,^25^ who reported staffing shortages in high‑volume centers and limited resources in smaller ones. Continued use of automated QA, predictive maintenance based on LSTM models, and flexible staffing arrangements (for example rotating physicists among facilities) will be necessary to keep pace with demand.

### 4.7 Study limitations and future considerations

Several factors limit the scope of this single‑institution, retrospective study and shape priorities for future work. First, confirmation in multi‑center settings is needed because local staffing, imaging protocols, and training datasets may influence the effect of AI contouring and other measures. Second, our analysis focused on workflow metrics rather than clinical end‑points; future studies should add longitudinal data on tumor control, toxicity, and patient‑reported quality of life. Third, timing data were entered manually in the MOSAIQ system, a method prone to small inaccuracies; real‑time electronic tracking would improve precision. Fourth, plan quality was not assessed with dosimetric indices, and equipment uptimes were not quantified, so subsequent projects should include dose‑volume metrics, conformity indices, and log‑based assessments of machine availability. Fifth, multiple internal and external factors—staffing levels, scheduling policies, and treatment volume among them—changed in parallel; prospective or multivariate designs will be needed to untangle their individual contributions.

To widen adoption, future research should test streamlined versions of these interventions in small or resource‑limited centers, perform cost‑benefit analyses, and encourage data sharing through multi‑center collaborations. Such efforts will help standardize best practices and support sustainable, efficient radiotherapy across diverse clinical environments.

## 5 CONCLUSIONS

This 4‐year follow‐up study on workflow optimization in radiotherapy demonstrates sustained improvements in operational efficiency, patient satisfaction, and treatment safety. The department successfully reduced workflow durations and significantly improved QA and patient‐centered care by integrating AI‐driven tools, standardized workflows, and data‐driven management systems. These advancements highlight the effectiveness of automation, predictive analytics, and structured process optimization in modern radiotherapy management.

The findings emphasize the transformative potential of AI‐assisted interventions in improving treatment planning efficiency and workflow coordination. Future research should focus on validating these interventions in multi‐center studies, particularly in resource‐limited settings, to assess scalability and adaptability across diverse healthcare environments. Additionally, addressing region‐specific barriers, including staff shortages and equipment constraints, will be critical for ensuring sustainable implementation. A broader and internationally informed approach to radiotherapy workflow optimization may help reduce disparities in access to efficient treatment, thereby supporting improved care delivery across diverse clinical settings.

## AUTHOR CONTRIBUTIONS

Chenlei Guo and Jianrong Dai designed the research. Chenlei Guo and Peng Huang contributed to the study concept. Chenlei Guo, Hao Jing, Zhuanbo Yang, and Bining Yang provided the study coordination and clinical support. Chenlei Guo and Wenlong Xia were involved in data analysis and wrote the manuscript. Kuo Men and Jianrong Dai provided the manuscript revision. All authors provided study materials or patient data and approved the final version of the manuscript.

## CONFLICT OF INTEREST STATEMENT

The authors declare no conflict of interest.

## ETHICS APPROVAL AND CONSENT TO PARTICIPATE

Ethics approval is not applicable for this type of study.

## CONSENT FOR PUBLICATION

Not applicable.

## Supporting information



Supporting information

## Data Availability

Research data are stored in an institutional repository and will be shared upon request to the corresponding author.
