# Supplementary material for: Longitudinal evaluation of workflow optimization in radiotherapy: A 4‐year retrospective study
Source: J Appl Clin Med Phys. 2025 Aug 31;26(9):e70252. doi: 10.1002/acm2.70252 (PMC12398953; doi:10.1002/acm2.70252)
Supplement: Supplementary file 1 — Supporting information [file ACM2-26-e70252-s001.docx]

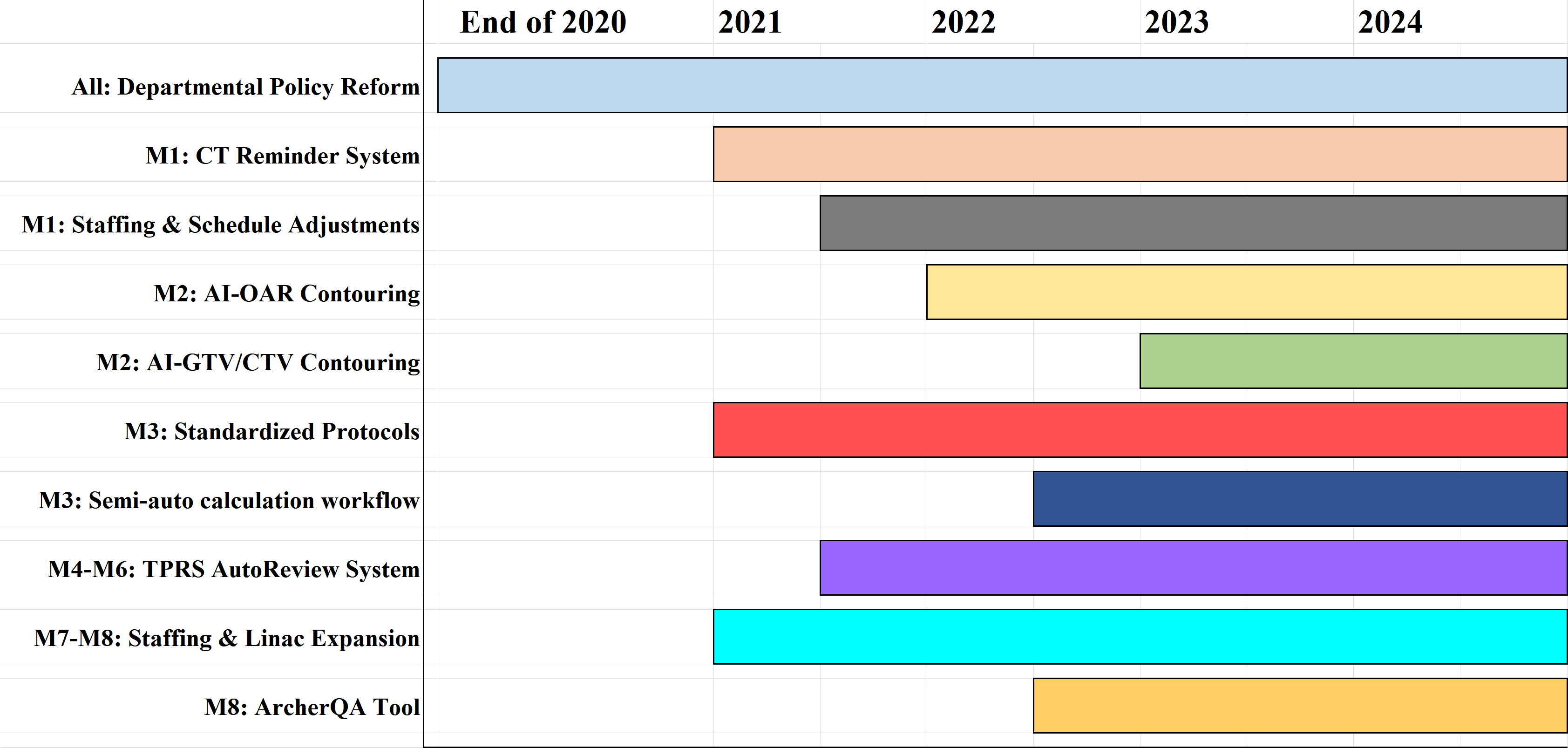


**Fig. S1.** Gantt-style timeline of workflow optimization interventions implemented in the radiotherapy department between late 2020 and 2024. Each horizontal bar represents the implementation and operational period of a specific intervention, categorized by its corresponding workflow module (M1-M8). The leftmost hatched segment of every bar denotes the initial “ramp‑up” or training phase, while the solid segment represents full clinical operation. The timeline captures departmental reforms, technical solutions (e.g., AI-assisted contouring, semi-automated calculation), and system-wide enhancements (e.g., automated plan review, staff and equipment expansion, quality assurance tools). The foundational policy reform initiated in late 2020 enabled the phased deployment and sustained execution of subsequent interventions.
